# Retinal Axonal Loss Begins Early in the Course of Multiple Sclerosis and Is Similar between Progressive Phenotypes

**DOI:** 10.1371/journal.pone.0036847

**Published:** 2012-05-23

**Authors:** Jeffrey M. Gelfand, Douglas S. Goodin, W. John Boscardin, Rachel Nolan, Ami Cuneo, Ari J. Green

**Affiliations:** 1 University of California, San Francisco Department of Neurology, Multiple Sclerosis Center, University of California San Francisco, San Francisco, California, United States of America; 2 Departments of Medicine and Epidemiology and Biostatistics, University of California San Francisco, San Francisco, California, United States of America; Charité University Medicine Berlin, Germany

## Abstract

**Background:**

To determine whether retinal axonal loss is detectable in patients with a clinically isolated syndrome (CIS), a first clinical demyelinating attack suggestive of multiple sclerosis (MS), and examine patterns of retinal axonal loss across MS disease subtypes.

**Methodology/Principal Findings:**

Spectral-domain Optical Coherence Tomography was performed in 541 patients with MS, including 45 with high-risk CIS, 403 with relapsing-remitting (RR)MS, 60 with secondary-progressive (SP)MS and 33 with primary-progressive (PP)MS, and 53 unaffected controls. Differences in retinal nerve fiber layer (RNFL) thickness and macular volume were analyzed using multiple linear regression and associations with age and disease duration were examined in a cross-sectional analysis. In eyes without a clinical history of optic neuritis (designated as “eyes without optic neuritis”), the total and temporal peripapillary RNFL was thinner in CIS patients compared to controls (temporal RNFL by −5.4 µm [95% CI −0.9 to −9.9 µm, p = 0.02] adjusting for age and sex). The total (*p* = 0.01) and temporal (*p* = 0.03) RNFL was also thinner in CIS patients with clinical disease for less than 1 year compared to controls. In eyes without optic neuritis, total and temporal RNFL thickness was nearly identical between primary and secondary progressive MS, but total macular volume was slightly lower in the primary progressive group (p<0.05).

**Conclusions/Significance:**

Retinal axonal loss is increasingly prominent in more advanced stages of disease – progressive MS>RRMS>CIS – with proportionally greater thinning in eyes previously affected by clinically evident optic neuritis. Retinal axonal loss begins early in the course of MS. In the absence of clinically evident optic neuritis, RNFL thinning is nearly identical between progressive MS subtypes.

## Introduction

Axonal loss is thought to be a major contributor to long-term disability in multiple sclerosis (MS). [1] Axonal loss is most conspicuous in the later stages of MS, [2], [3] but MR imaging studies suggest that more widespread axonal injury probably begins much earlier in the disease course. At the time of a first clinical relapse (a clinically isolated syndrome or CIS), CIS patients tend to have smaller gray matter volumes on magnetic resonance imaging (MRI) [4], [5] lower brain N-acetyl aspartate levels (a spectroscopic marker of neuroaxonal injury) [6] and greater whole brain atrophy on MRI [7], [8] compared to controls without MS. Patients with early relapsing-remitting MS also exhibit lower gray and white matter fractional volumes on MRI than unaffected controls. [9] Such MRI findings may not be entirely attributable to axonal loss, however, as brain atrophy could reflect loss of non-neuronal cells (which constitute about half of all cells in the human brain and over two-thirds of cells in human cortical white matter), [10] neocortical demyelinating lesions can potentially confound segmentation algorithms, [11], [12] and low NAA levels can indicate reversible axonal dysfunction in the absence of frank axonal degeneration. [13]

The retina provides an attractive site for assessing axonal loss in MS, [3], [14] as the retinal nerve fiber layer (RNFL) is composed almost entirely of axons. Retinal nerve fiber layer thickness can be quantified using optical coherence tomography (OCT), a non-invasive technique in which the backscatter of infrared light is used to generate cross-sectional images. [15], [16] Previous studies in MS have demonstrated thinning of the RNFL and a reduction in macular volume, most prominently in eyes previously affected by optic neuritis. [3], [14], [17], [18], [19], [20], [21], [22], [23], [24] RNFL thinning in MS appears to progress over time, [25], [26] and is associated with greater disease severity [27] and greater cortical gray matter atrophy. [28]

While brain imaging measures suggest that axonal injury occurs early in the course of MS, including in patients with a CIS, [4], [7] previous work using time-domain OCT did not detect retinal axonal loss in patients with a CIS. [29], [30] There are at least three possible explanations for this apparent inconsistency between brain MRI and retinal OCT: 1) retinal pathology in MS may not reflect pathology in the rest of the brain; 2) MRI metrics may be confounded by involvement of non-neuronal structures; and/or 3) the lower spatial resolution of earlier-generation time-domain OCT methods may have made it harder to detect differences in retinal thickness early in the disease course. Newer spectral-domain (SD)-OCT techniques enable faster image acquisition and improved image registration, permitting greater reproducibility and accuracy. [31]

We hypothesized that retinal axonal loss occurs early in the course of MS. In this cross-sectional analysis, we examined whether RNFL thickness measured using SD-OCT differs between patients with high-risk CIS and unaffected controls.

Another question that remains unsettled in the literature is whether retinal axonal thinning differs between patients with the primary progressive and secondary progressive phenotypes of MS. Some previous studies using time-domain OCT found no significant RNFL thinning in PPMS compared to patients with relapsing MS, [19], [24] while another study reported prominent RNFL thinning and macular volume loss in both primary and secondary progressive MS. [20] In this analysis, we also examined whether SD-OCT patterns of RNFL thinning differ between patients with primary progressive and secondary progressive MS. Finally, point estimates of RNFL thickness in MS vary greatly in the literature, [17], [32] making it challenging to calculate the sample sizes necessary when using OCT as an outcome in clinical trials of neuroprotective and neurorestorative therapies. For this reason, we also examined how SD-OCT measures of retinal axonal thickness differ by disease stage and subtype.

In summary, the two major questions we examine using this large cross-sectional dataset of retinal SD-OCT measures in MS are: 1) Is RNFL thinning detectable in patients with a CIS, the first clinical stage of MS?, and 2) Does retinal axonal loss differ between the primary progressive and secondary progressive phenotypes of MS?

## Methods

### Study Population

All patients age 18 and older with a high-risk CIS or MS (by 2005 International Panel Criteria) [33] imaged using SD-OCT at the UCSF MS center between January 2008 and October 2011 were considered for inclusion in this study. Patients were excluded from analysis if a disease other than MS better explained their symptoms or if there was a history of glaucoma, diabetes, uveitis, age-related macular degeneration, retinal disease, severe myopia (as measured by a −6 or stronger prescription) or a cataract significant enough to affect OCT quality. Unaffected controls were recruited from the community and included some spouses and friends of patients with MS. All participants provided written informed consent, and the UCSF Committee on Human Research approved the study protocol.

MS stage and subtype (CIS, RRMS, SPMS and PPMS) were established by the treating MS specialist and confirmed by study investigators (JMG and AJG) through chart review. A CIS was defined as a first monosymptomatic clinical demyelinating attack typical of MS without evidence of dissemination in time. [33] In order to enrich the CIS group for patients at highest risk of progressing to MS, [34] CIS patients with at least one T2 hyperintensity typical of demyelination on conventional brain MRI were considered to be “high-risk” and were included for analysis. [34] A prior episode of symptomatic demyelinating optic neuritis was diagnosed when there was a history of a subacute episode of visual blurring or visual loss associated with eye pain and when this event was confirmed by medical record review and subject interview. Age was measured at the time of the OCT evaluation. Disease duration was defined as the time from the first clinical symptom attributable to MS to the SD-OCT examination. The Expanded Disability Score Scale (EDSS), [35] was determined by the treating MS specialist and confirmed by the study investigators through record review.

### Visual Evaluations

High contrast visual acuity was measured using a computerized Early Treatment Diabetic Retinopathy Study (ETDRS) chart (ProVideo system, INNOVA Systems, Burr Ridge, Illinois) and analyzed using the logarithm of the Minimum Angle of Resolution (LogMAR) scale. Low contrast vision was assessed using a computerized chart (ProVideo system) at 20/200 measuring the lowest contrast level at which subjects could read letters. Scoring for low contrast vision was assigned on a 5 to 100 point scale with 5 points given for every step-up in low-contrast ability (i.e. 100 points for reading at 1.2% contrast and 5 points for best reading ability at 100% contrast). Color vision was assessed using Hardy-Rand-Rittler plates (scored as the number correct out of 19 plates).

### Spectral-Domain Optical Coherence Tomography

SD-OCT was performed using the Spectralis OCT platform (Heidelberg Engineering, Heidelberg, Germany), which performs up to 40,000 A scans per second using an 870 nm bandwidth light source. SD-OCT retinal thickness scans were obtained by a trained technician and replicated three times by a single operator within a single session. Correction for spherical errors was adjusted prior to each measurement. The peripapillary RNFL was measured at a distance of 3.4 mm from the center of the papilla. For peripapillary B-scans, 0 degrees was defined as the nasal-most edge of the disc. The nasal quadrant RNFL was defined as the region between 315 and 45 degrees and the temporal quadrant RNFL as the region between 135 and 225 degrees. For macular volume measurements, 20×15 degree raster scans were performed consisting of 19 high-resolution line scans. Scans with insufficient signal to noise or edge detection or retinal thickness algorithm failure were excluded and measurements were repeated until good quality was achieved.

### Statistical Analysis

Multiple linear regression was used to examine differences in RNFL thickness (total RNFL, temporal RNFL and nasal RNFL) and macular volume between groups, adjusting for age and sex. To account for possible inter-eye correlations when two eyes from the same patient were included in the model, the standard error was adjusted using the clustered sandwich estimator. Possible modification of the association between age or disease duration and retinal thickness outcomes by disease subtype was assessed by including interaction terms for age or disease duration with disease subtype in the regression models. A p-value of 0.05 or less was considered to be statistically significant. Statistical analyses were performed using Stata 12 (StataCorp, College Station, TX). Figures were generated using GraphPad Prism 5.

## Results

### Demographics

541 patients with MS or CIS met inclusion criteria, including 45 patients with high-risk CIS; 403 with relapsing-remitting (RR)MS; 60 with secondary-progressive (SP)MS; and 33 with primary-progressive (PP)MS. Within the high-risk CIS group, 22 patients had a clinical disease duration of less than 1 year (40 eyes were studied in this group) and 8 of the CIS patients presented with optic neuritis as the first clinical symptom. A comparison group of 53 unaffected controls was also studied. Table 1 lists baseline demographic and clinical information for each patient population. Table 2 lists retinal SD-OCT measures categorized by MS stage and subtype and optic neuritis history, together with measures of high and low contrast visual acuity and color vision testing.

**Table 1:** Demographics.

	Controls	Clinically Isolated Syndrome	Relapsing-Remitting MS	Secondary Progressive MS	Primary Progressive MS
	(N = 53)	(N = 45)	(N = 403)	(N = 60)	(N = 33)
**Age** Mean (SD)	34.6 (11.1)	39.3 (10.2)**	42.3 (11.1)**	51.4 (10.7)**	52 (11.8)**
**Sex** (% Female)	57	80**	72*	68	45
**Disease Duration** Median (IQR) in years	–	1 (0.3–2.5)	6.7 (2.7–12.1)	13.5 (6.4–21.2)	8.6 (4–11.7)
**Expanded Disability Status Scale (EDSS)** Median (IQR)	–	1.5 (1–2)	2 (1.5–3.5)	5.5 (4–6.5)	5.5 (4–6.5)

* *p*<0.05;

** *p*<0.01 – Statistical differences refer to comparison with unaffected controls.

**Table 2:** RNFL Thickness and Macular Volume by MS Stage and Subtype.

	Controls	Eyes Without Prior Optic Neuritis	CIS	RRMS	SPMS	PPMS	Eyes with Prior Optic Neuritis	CIS	RRMS	SPMS
	106 Eyes		82 Eyes	569 Eyes	103 Eyes	66 Eyes		8 Eyes	238 Eyes	16 Eyes
**High-Contrast Visual Acuity** (LogMAR)	−0.15 (.11)		−0.1 (.16)	−0.06 (.15)**	.01 (.21)**	0.08 (.3)*		−0.09 (.12)	0.06 (.31)**	0.26 (.44)*
**Low Contrast Vision** (0–100 Scale)	91.4 (7.6)		88.6 (14.9)	88.5 (12.8)*	83.5 (16.2)*	84.8 (17.1)		84.8 (9.9)*	75.2 (26.9)**	71.3 (21.6)*
**Color Vision** (HRR Plates) (0–19 Scale)	17.7 (2.2)		17.3 (1.7)	16.6 (3)	14.3 (5.1)*	12.5 (5.9)*		15.9 (2.9)	13.4 (5.6)**	7.5 (6.6)**
**Total RNFL** Mean (SD) µm	101.3 (10.1)		98.2 (8.4)*	92.9 (13)**	85.5 (14.3)**	80.5 (15.4)**		91.6 (8.5)**	80.3 (18.1)**	71.7 (13.2)**
**Temporal RNFL** Mean (SD) µm	74.5 (11.4)		70 (12.2)*	63 (14.2)**	57.1 (13.2)**	53.5 (14.7)**		59 (15.2)*	50.9 (15.1)**	42.8 (14.8)**
**Nasal RNFL** Mean (SD) µm	76.7 (15.8)		75.4 (13.4)	73 (16.1)	70.1 (17.1)	63.7 (16.3)*		72.6 (9.7)	63.2 (18.1)**	58.7 (10.2)**
**Macular Volume** Mean (SD) mm^3^	3.14 (0.12)		3.09 (.15)	3.05 (.16)**	3.01 (.19)*	2.92 (.21)*		3.01 (.16)*	2.93 (.17)**	2.87 (.16)**

* *p*<0.05;

** *p*<0.001. Statistical differences refer to comparison with healthy controls using linear regression to adjust for age and sex. The standard error was adjusted for possible intra-patient inter-eye correlations. CIS = Clinically Isolated Syndromes, RRMS = Relapsing-Remitting Multiple Sclerosis, SPMS = Secondary Progressive Multiple Sclerosis, PPMS = Primary Progressive Multiple Sclerosis; RNFL = Retinal Nerve Fiber Layer.

### Retinal Nerve Fiber Layer Thinning is Detectable in Patients with a Clinically Isolated Syndrome, the Earliest Clinical Stage of MS

In the eyes of CIS patients without prior symptomatic optic neuritis, the total RNFL was thinner compared to unaffected controls, and was especially thin in the temporal peripapillary region (the temporal RNFL was thinner by 5.4 µm [95% CI −0.9 to −9.9 µm, p = 0.02] adjusting for age and sex). There was no difference in macular volume between CIS patients and controls. The total and temporal RNFL were thinner in the subgroup of CIS patients with clinical disease for less than one year compared to controls (the total RNFL was 6 µm thinner than controls [95% CI −1.5 to −10.6 µm, p = 0.01] and the temporal RNFL was 6.3 µm thinner than controls [95% CI −0.7 to −11.9, *p* = 0.028, adjusting for age and sex). There were no differences in total, temporal or nasal RNFL thickness or macular volume between the eyes of CIS patients without optic neuritis and fellow eyes of CIS patients with a history of unilateral optic neuritis. Follow-up information about disease activity was available for 36 of the CIS patients – 7 were subsequently diagnosed with relapsing-remitting MS by International criteria. [33] There were no differences in baseline total, temporal or nasal RNFL thickness or total macular volume between CIS patients who subsequently developed RRMS and those who remained categorized as CIS when analyzing all eyes or restricting the analysis to eyes without prior symptomatic optic neuritis.

### RNFL Thickness Is Similar in Primary and Secondary Progressive MS in Eyes Without Prior Optic Neuritis

**Figure 1:**
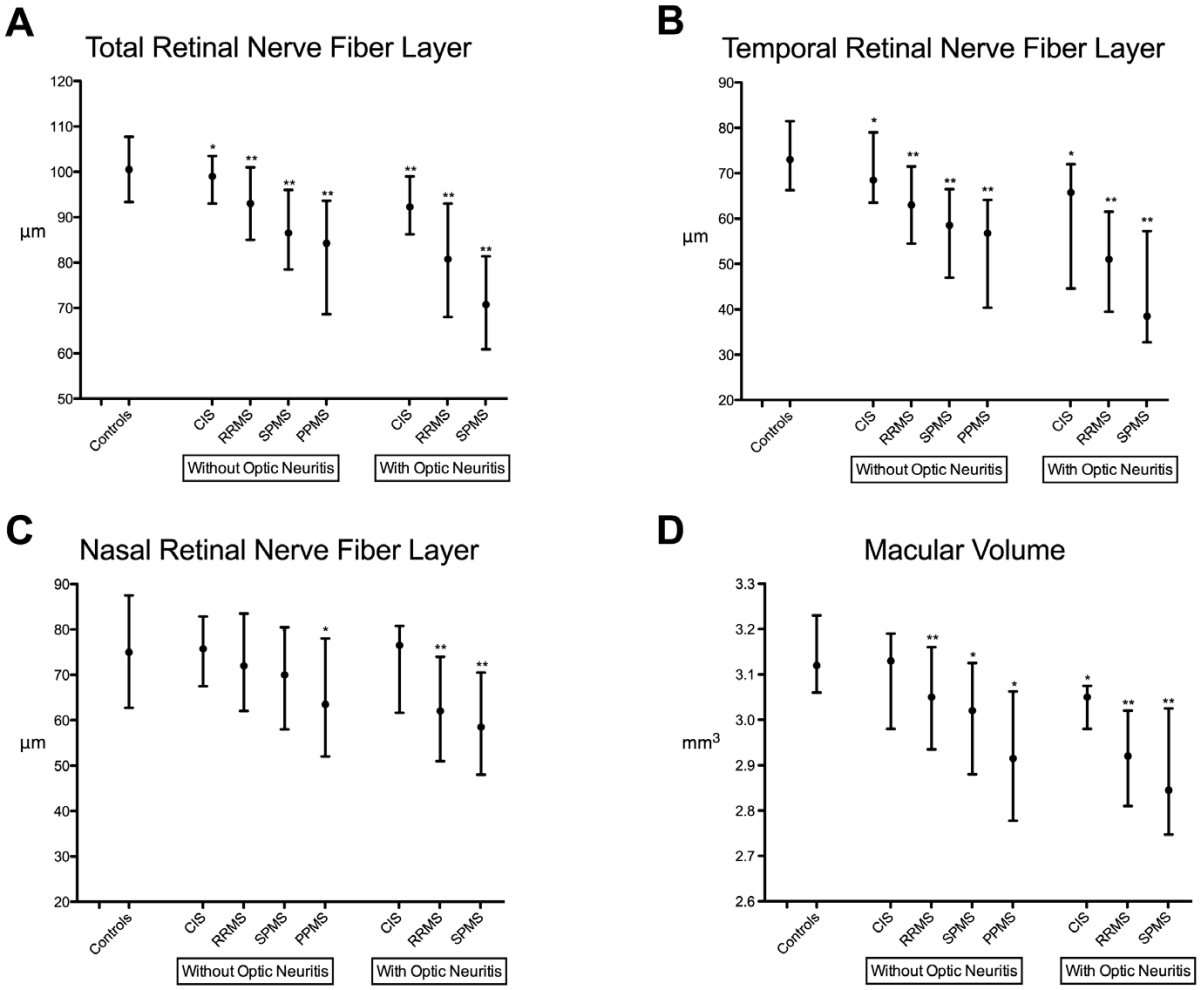
Retinal Axonal Degeneration in Multiple Sclerosis is Increasingly Prominent in More Advanced Stages of Disease and Proportionally Greater in Eyes Previously Affected by Symptomatic Optic Neuritis. Retinal Nerve Fiber Layer thickness (A, B, C) and macular volume (D), as measured by spectral-domain optical coherence tomography (Heidelberg Spectralis) in a cross sectional sample of patients with high-risk Clinically Isolated Syndromes (CIS) (n = 45), Relapsing-Remitting MS (RRMS) (n = 403), Secondary-Progressive MS (SPMS) (n = 60), Primary-Progressive MS (PPMS) (n = 33) and unaffected controls (n = 54). Both the total and temporal peripapillary RNFL were thinner in CIS patients compared to controls in eyes without prior symptomatic optic neuritis. RNFL measures were nearly identical between SPMS and PPMS patients in eyes without optic neuritis, but macular volumes were lower in PPMS compared to SPMS patients in eyes without optic neuritis (p<0.05). The black dots denote the median, and the bars signify the interquartile range. *p<0.05, **p<0.001 refers to the comparison with unaffected controls using linear regression to adjust for age and sex.

**Figure 2:**
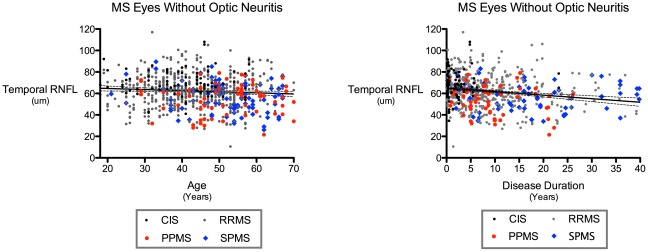
Associations of Temporal Retinal Nerve Fiber Layer Thickness by Disease Stage and Subtype with Age and Disease Duration in MS. Temporal quadrant peripapillary retinal nerve fiber layer (RNFL) thickness by age (A) and disease duration (B) in MS patients in eyes without a history of symptomatic optic neuritis. Note that temporal RNFL thickness is nearly identical between patients with primary and secondary progressive MS, but disease durations tend to be greater in SPMS and shorter in PPMS for the same degree of RNFL loss. *The solid line indicates the slope as fitted by linear regression, and the dotted lines denote 95% confidence intervals. CIS = Clinically Isolated Syndrome; RRMS = Relapsing-Remitting MS; PPMS = Primary Progressive MS; SPMS = Secondary Progressive MS.*

There was no meaningful difference in age between the PPMS and SPMS groups (p = 0.89). RNFL thickness was clinically indistinguishable between patients with PPMS and SPMS in eyes without a history of symptomatic optic neuritis (see Figure 1 and Tables 1 and 2). Color-coded scatter plots of temporal RNFL thickness by age (Figure 2A) and disease duration (Figure 2B) illustrate how PPMS and SPMS non-optic neuritis eyes exhibit similar amounts of RNFL loss, but how this occurs earlier in the clinical duration of disease in patients with PPMS. Total macular volumes were lower in patients with PPMS compared to SPMS in eyes without optic neuritis (the macular volume was 0.10 mm^3^ lower in the PPMS group [95% CI .002–0.19 mm^3^, p = 0.046, adjusting for age and sex]).

### Temporal-Predominant RNFL Thinning in MS is Most Pronounced In Advanced Stages of Disease, Even in Eyes Previously Affected by Symptomatic Optic Neuritis

In general, compared to controls, eyes with a prior history of optic neuritis demonstrated thinning of the retinal nerve fiber layer, most conspicuously and severely in the temporal quadrant, which contains fibers of the papillomacular bundle (Figure 1, Tables 1 and 2). A similar pattern of temporal-predominant peripapillary RNFL thinning was observed across MS subtypes in eyes without prior symptomatic optic neuritis. RNFL thinning was increasingly prominent in patients with more advanced stages of the disease – SPMS>RRMS>CIS – and most pronounced in eyes previously affected by symptomatic optic neuritis – SPMS optic neuritis>RRMS optic neuritis>CIS optic neuritis. This gradation persisted after adjusting for cumulative number of optic neuritis episodes. This indicates that even in eyes previously affected by symptomatic optic neuritis, advanced disease stage is associated with proportionally greater retinal axonal thinning. There was no apparent modification of the association between age or disease duration and temporal RNFL thickness by disease subtype.

## Discussion

This study demonstrates that 1) retinal axonal thinning begins early in the course of MS and independently of the occurrence of symptomatic optic neuritis and 2) that in the absence of symptomatic optic neuritis, RNFL thickness is nearly identical between progressive MS subtypes.

Previous studies using time-domain OCT to examine retinal axonal degeneration in CIS found no differences in retinal nerve fiber layer thickness or macular volumes between CIS patients and controls. [29], [30] This may reflect differences in study populations, differences in methodology or the lower spatial resolution of the time-domain OCT technique used in these previous studies. Other studies have reported greater retinal axonal thinning in patients with MS compared to patients with CIS. [21], [36]

Temporal-predominant peripapillary retinal nerve fiber layer thinning is characteristic in MS, [3], [19], [20], [27], [37] and the prominence of this pattern of RNFL thinning in CIS patients indicates that temporal-predominant RNFL loss begins early in the disease course. The cause of temporal-predominant RNFL thinning in MS is unknown. The RNFL is made up primarily of retinal ganglion cell axons. There are three main types of retinal ganglion cells that synapse in the lateral geniculate nucleus – 1) smaller parvocellular cells, which are distributed overwhelmingly in the macula, 2) larger magnocellular cells, which are distributed primarily in the retinal periphery, and 3) koniocellular cells, which are distributed more diffusely and sparser in number. [38] On the standard peripapillary OCT scan, the RNFL temporal to the optic disc consists primarily of parvocellular axons within the papillomacular bundle that subserve central vision. [38] Autopsy studies of the lateral geniculate nucleus in people who died with late stage MS have demonstrated a selective loss of parvocellular (smaller-sized) axons in the lateral geniculate nucleus in MS with relative preservation of magnocellular (larger-sized) axons. [39] Autopsy studies of the spinal cord in patients who died with MS have also revealed a loss of smaller-sized axons in the lateral cortical spinal tracts of the cervical and thoracic spinal cord, with relative preservation of larger-sized axons. [40] Whether smaller sized axons are more vulnerable to injury in MS is unknown. It is also possible that smaller sized axons may remyelinate less efficiently than larger sized axons following demyelinating injury, given evidence from *in vitro* models of remyelination in which axonal scaffold size appears to be a critical determinant of oligodendrocyte precursor cell differentiation. [41] More research is needed to understand the cause of temporal-predominant thinning in MS.

In the absence of prior symptomatic optic neuritis, RNFL thickness was nearly identical between patients with progressive MS subtypes. These results differ from previous studies using time-domain OCT that found no significant retinal thinning in PPMS [19] and no difference in retinal thickness between PPMS and other types of MS [24], and are consistent with the findings of another study using time-domain OCT that found prominent RNFL and macular volume loss in progressive MS. [20] These results add to the mounting evidence of phenotypic similarities between PPMS and SPMS. Measures of genetic susceptibility to MS are similar between patients with primary and secondary progressive phenotypes of disease, [42] as are measures of global brain tissue damage and magnetization transfer imaging. [43] The median age at time of disease progression was also indistinguishable between primary and secondary progressive MS patients in a large French population-based study, as was the time it took to reach major disability milestones. [44] In our study, macular volumes were slightly lower in patients with primary MS compared to eyes without prior ON in secondary progressive MS patients. One possible interpretation of this result is that there may be a proportionally greater loss of other neuronal elements in the inner and outer retina in primary progressive disease. Future studies using segmentation algorithms will be helpful in exploring this possibly further.

Strengths of our analysis include the large sample size, which allows for more reliable point estimates for the observed values of retinal thickness at different stages of MS and the higher spatial resolution of the spectral domain OCT technique used in this study compared to time-domain OCT techniques used in some previous studies. [31] While the cross-sectional study design precludes comparison of differential rates of change over time across MS subtypes, it is also advantageous for examining associations with retinal thickness and disease stage over the lifetime of disease, as MS typically evolves over decades. One possible limitation of our analysis is that the control group was slightly younger than the disease group, which could bias the results, although we attempted to adjust for age using regression models.

This study demonstrates that retinal axonal thinning is detectable in patients with a CIS; that retinal thinning is nearly identical in patients with primary and secondary progressive MS in eyes without prior symptomatic optic neuritis; and that RNFL thinning is increasingly prominent in more advanced stages of disease, even in eyes with prior symptomatic optic neuritis. These findings support the possible utility of OCT as a marker of axonal injury for trials of neuroprotective and neurorestorative therapies in MS and support the idea that prevention of axonal injury is relevant from the earliest clinical stages of disease.
